# Sex- and Gender-specific Observations and Implications for COVID-19

**DOI:** 10.5811/westjem.2020.4.47536

**Published:** 2020-04-10

**Authors:** Lauren A. Walter, Alyson J. McGregor

**Affiliations:** * University of Alabama at Birmingham, Department of Emergency Medicine, Birmingham, Alabama; † Warren Alpert Medical School of Brown University, Department of Emergency Medicine, Providence, Rhode Island

This is a critical time for medicine. As we observe the exponential rise in the number of individuals in the United States (US) who are infected with COVID-19, we try to prepare. Those in the front lines are trying to protect themselves and their patients with the daily ration of personal protective equipment and ventilation assistive equipment. Many individuals are racing against time to develop the needed novel treatments and vaccines. Public health officials work with what little information is known in order to make effective recommendations for prevention. However, at this pivotal time in history where every detail obtained by US health officials could be lifesaving, we are leaving out vital information.

Descriptive and observational data from Wuhan, China, note that the majority (51%–66.7%) of affected patients have been male. In addition, male sex was an independent risk factor associated with refractory disease and death (2.8% death rate for men vs 1.7% for female).^1^,^2^ Currently, men represent 58% of COVID-19 infected patients in Italy and 70% of COVID-related deaths.^3^ As coronavirus cases and deaths in the US continue to soar, sex-specific, comprehensive data with regard to US patients is not yet available.

Sex- and gender-based medicine (SGBM) incorporates how biological *sex* and the sociocultural aspects of *gender* affect health and illness. It acknowledges the interrelationship between sex and gender on health outcomes and promotes consideration of this variable in both research and clinical practice. SGBM has demonstrated significant evidence-based impact on cardiovascular disease, stroke, sports medicine, and pain management, just to name a few

Sex and gender differences have been observed in infectious diseases previously. On a broad and critical scale, Nasir et al demonstrated that males with all-cause infectious sepsis have a 70% greater mortality than their female counterparts. Likewise, respiratory infection-specific epidemiological data from recent SARS (2003) and MERS (2012) outbreaks demonstrated a significantly higher case fatality rate in males as compared to females, 21.9% vs 13.2%.^4^,^5^

## Sex-specific Factors

Is the biological male more susceptible to an increased severity of infection? Or does the biological woman have natural protection against these viruses? In a 2017 *BMJ* article, Dr. Kyle Sue demonstrated the effect of sex hormones, estrogen and testosterone, on immune system response and engagement, resulting in a less robust immunologic response in males and subsequent increased morbidity and mortality from viral respiratory illnesses.^6^ In addition, the X chromosome carries the largest number of immune-related genes in the human genome, perhaps also contributing to female’s superior immune response (as well as a female preponderance in autoimmune diseases).^7^

Angiotensin-converting enzyme 2 (ACE2) and its role in viral transmission and associated morbidity has also been a topic of recent COVID-19 associated discussion. ACE2 receptors on pulmonary endothelium serve as a main entry point for coronavirus. Several previous animal models have demonstrated increased ACE2 activity in the male or ovariectomized model, suggesting a sex hormone influence.^8^ The gene for the ACE2 receptor is also, interestingly, on the X chromosome.^9^

## Gender-specific Factors

Behavioral and cultural variables have also influenced current COVID-19 epidemiology. Smoking in particular has been implicated as a significant contributor to disease severity, and gender-specific patterns are quite apparent here. The smoking rate in China is much higher in men than in women (288 million men vs 12.6 million women; 2018 data).^10^ Likewise, in Italy, men are more likely to smoke than women at any age (1.12x to 1.7x, depending on age cohort; 2018 data).^11^ Similar gender-specific trends are also present in the US, where 17.6% of smokers are men as compared to 13.6% of women.^12^

In addition, as the traditional caregivers and coordinators of care for their loved ones, women, particularly working mothers, tend to spend more time than men focused on medical issues related to both their own healthcare and that of their families.^13^ In general, men are more likely to engage in health-related risks which, even prior to the COVID-19 pandemic, has been shown to result in higher rates of injury and disease.^14^ Suen et al demonstrated in 2019 that being a middle-aged female was a protective factor with regard to hand hygiene knowledge and practice.^15^

## Implications for COVID-19 Management

As clinical researchers and pharmaceutical companies race to find an effective treatment strategy or vaccine for COVID-19, no sex- or gender-specific recommendations have been released with regard to the care and management of individuals affected by the novel coronavirus. Appreciating the weight of known sex- and gender-specific epidemiologic observations thus far, however, will be an important highlight of the information gathered to date. This, combined with what is already known about sex- and gender-based pulmonary and infectious disease pathology, may lead to important treatment breakthroughs that consider the sex and/or gender of patient in the comprehensive management plan.

In addition, the current pandemic weighs heavily on emotional wellness along with physical health. COVID-19 has also released a contagion of fear, anxiety, and stigma that will have implications for downstream mental health effects including post-traumatic stress disorder (PTSD). In general, the prevalence of PTSD has been shown to be substantially higher in women.^16^ This has been re-substantiated in the setting of the COVID-19 outbreak in Wuhan, China, where women scored significantly higher on the PCL-5 (DSM-5 self-report measure for PTSD) than men, including higher rates of re-experiencing and negative alterations in cognition or mood.^17^ Early recognition and effective treatment can ameliorate the burden of PTSD on both the individual and society, particularly for women who have been shown to have a modest advantage with regard to treatment response.^18^

## Future Considerations

Since 2016, the NIH has required inclusion of sex as a biological variable (SABV) in the study design for funded research.^19^ Recognizing the weight these variables play in disease outcome should result in universal adoption of SABV as scientists develop and engage in COVID-19 research. While men appear to be disproportionately affected and at risk for COVID-19 infection and associated morbidity, researchers should avoid the slippery slope of the traditional male-dominant test and analysis approach.

**Population Health Research Capsule**

What do we already know about this issue?

COVID-19 represents an unparalleled public health crisis. Like many other infectious diseases, sex and gender differences in health outcomes have already been globally observed.

What was the research question?

We sought to summarize and explain known COVID-19-related sex and gender differences.

What was the major finding of the study?

Sex and gender differences are having significant impacts on current COVID-19 health outcomes.

How does this improve population health?

This perspective brings attention to the importance of sex and gender, specifically as they impact current clinical management and research during the COVID-19 pandemic.

When considering pharmaceutical therapy advances, several previous studies have established that women are much more likely to experience adverse drug reactions (ADR) than men.^20^ In fact, historically the majority of drugs recalled from the market were done so due to serious ADRs reported by women, quite often because they were never tested on women during clinical trials. Several sex-specific pharmacokinetic and pharmacodynamic differences have been well documented.^21^

Yes, time is of the essence right now; however, COVID-19 does not get a “pass” in considering sex and gender when gathering data or testing treatments. Sex and gender have already proven to be crucial variables in the short history of COVID-19; they will continue to be factors of marked importance. Making healthcare providers and researchers aware of their impact in real time will be crucial to the integration of susceptibility profiles and improving outcomes during an active public health crisis.
